# Supplementary material for: Insights into silicon cycling from ice sheet to coastal ocean from isotope geochemistry
Source: Commun Earth Environ. 2025 Apr 19;6(1):305. doi: 10.1038/s43247-025-02264-7 (PMC12009215; doi:10.1038/s43247-025-02264-7)
Supplement: Supplementary file 2 — supplementary information [file 43247_2025_2264_MOESM2_ESM.pdf]

Insights into silicon cycling from ice-sheet to coastal ocean from isotope geochemistry

Supplementary Information

Katharine R. Hendry[1,2], Felipe Sales de Freitas[3], Sandra Arndt[3], Alexander Beaton[4], Lisa Friberg[2], Jade E. Hatton[2,5,6], Jonathan R. Hawkings[7], Rhiannon Jones[1], Jeffrey W. Krause[8,9], Lorenz Meire[10,11], Hong Chin Ng[2], Helena Pryer[12], Sarah Tingey[13], Sebastiaan J. van de Velde[14,15,16], Jemma Wadham[13], Tong Wang[2], E. Malcolm S. Woodward[17]

[1] British Antarctic Survey, High Cross, Madingley Road, Cambridge, CB3 0ET, UK

[2] School of Earth Sciences, University of Bristol, Queen's Road, Bristol, BS8 1RJ

[3] BGeosys, Department of Geosciences, Université libre de Bruxelles, Brussels, Belgium

[4] National Oceanography Centre Southampton, European Way, Southampton, SO14 3ZH, UK

[5] Department of Ecology, Faculty of Science, Charles University, Viničná 7, Prague 2, 128 44, Czechia

6 UK Centre for Ecology & Hydrology, Environment Centre Wales, Bangor, UK

[7] Department of Earth and Environmental Science, University of Pennsylvania, 240 South 33rd Street, Philadelphia, PA 19104, USA

[8] Dauphin Island Sea Laboratory, 101 Bienville Boulevard, Dauphin Island, AL 36528, USA

[9] Stokes School of Marine and Environmental Sciences, University of South Alabama, Mobile, AL 36688, USA

[10] Greenland Climate Research Centre, Greenland Institute of Natural Resources, Nuuk, Greenland

[11] Department of Estuarine and Delta Systems, Royal Netherlands Institute for Sea Research, Yerseke, The Netherlands

[12] Department of Earth Sciences, University of Cambridge, Downing Street, Cambridge, CB2 3EQ, UK

[13] iC3: Centre for ice, Cryosphere, Carbon and Climate, Department of Geosciences, UiT The Arctic University of Norway, 9037 Tromsø, Norway.

[14] Department of Biology, University of Antwerp, Wilrijk, Belgium

[15] Department of Marine Science, University of Otago, Dunedin, New Zealand

[16] National Institute of Water and Atmospheric Research, Wellington, New Zealand

[17] Plymouth Marine Laboratory, Prospect Place, The Hoe, Plymouth PL1 3DH, UK

Supplementary Notes S1: Summary of sequential sediment extractions

Reactive silica phases can be sequentially extracted from sediments using a series of chemical treatments. Extracted 'pools' of silica are 'operationally-defined', i.e., are defined entirely based on their chemical reactivity rather than by specific components. The extracted

solutions are analysed for DSi, trace elements (using ICP-OES or ICP-MS) and stable silicon isotopes.

The extraction generally used is based on [1,2] and is as follows:
(1) mild acid-leachable silica (Si-HCl) is extracted using 0.1 M HCl at room temperature for 18 hours; this phase is consistently isotopically light (and has been measured in systems ranging from low-latitude river plumes to high-latitude basins) and often associated with high levels of Fe, Mn and Al and is thought to have a significant contribution from Si adsorbed onto Fe and Mn phases[1,3-5]. Note that in some studies an additional hydrogen peroxide leach is used to remove organics e.g., [3,5,6].

(2) mild alkaline leachable amorphous silica (Si-Alk) is extracted using 0.1 M Na_2CO_3 at 85°C, often using a time-series approach similar to [7]; this fraction likely includes BSi and glacier-derived ASi. This pool is usually isotopically heavier than the other fractions, especially if most of the pool is from water-column diatoms.

(3) in some studies a strong alkaline leach is also used to extract the most reactive crystalline phases[1,3]. This pool usually has a 'crustal' $\delta^{30}Si$ signature (i.e., ~ 0‰).

Whilst there are differences in the methodologies between laboratories, in the situations where there are two different groups that have studies glacial sediments from a similar location, the bulk ASi data are in broadly good agreement e.g., Svalbard e.g., this study and [8].

Supplementary Figure S1: Sequential extraction results for different sediment types among various glaciated environments, all carried out with the same methodology (See above). The blue lines show reactive silica extracted using a weak acid leach (Si-HCl); the red lines show reactive silica extracted using a weak alkaline heating extraction (Si-Alk). Data are from [4,5]. Data from Iceland and Svalbard are new to this study (see Supplementary Dataset).

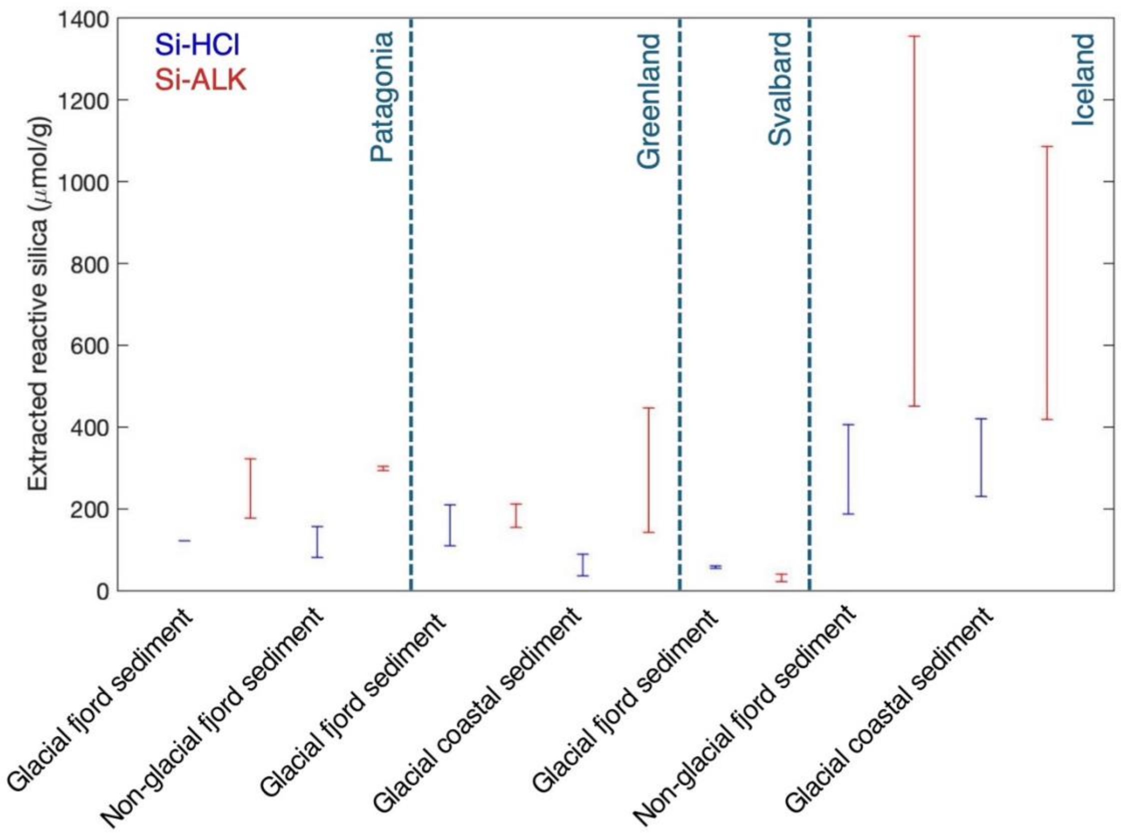

Supplementary Methods M1: Silicon(-metals) dynamics along glaciated fjords – a hypothetical fjord system RTM exercise

Rationale
Several lines of evidence suggest a close link between silicon and metals cycling in glaciated environments e.g., [4]. Critically, such dynamics modulate particulate silicon dissolution, precipitation and burial, dissolved silicon (DSi) accumulation in porewaters and return fluxes to bottom waters.
We hypothesise that glacial amorphous silicon (ASi) [and reactive metals] supply can largely modify fjord sedimentary silicon dynamics. Thus, to test and quantify such hypothesis, we designed a model experiment which simulates benthic silicon processing in a hypothetical fjord system transect from fjord head to fjord opening (see main text and Fig. 5). We based our system on *typical* glaciated fjord conditions.

Model set up
We ran the model for three idealised stations along a generic transect for our hypothetical fjord system (see main text and Fig. 5). Core A represents the innermost, strongly ASi influenced site. Core B sits on an intermediate position along the transect. Core C represents the outermost, strongly influenced by pelagic conditions and BSi deposition. We made the following assumptions when designing the model runs for the hypothetical fjord system (see Tables T1–T3 for details):

(i) The fjord head receives greater ASi supply from glacial sources and the ASi input attenuates downstream along the transect.
(ii) The ASi turbidity plume impacts BSi pelagic production. As such, BSi pelagic production remains low at the fjord head and progressively increases towards the fjord opening.
(iii) DSi bottom water concentrations remain invariant along the transect.
(iv) Iron-adsorbed silicon (FeSi) occurs as a pre-formed silicon phase in the water column. The FeSi supply display greater input at the fjord head and attenuates along the transect.
(v) FeSi continuously forms in the upper, oxic sediment layers.
(vi) FeSi releases DSi to porewaters in the lower, suboxic/anoxic sediments layers due to iron oxyhydroxide reductive dissolution.
(vii) ASi and BSi undergo dissolution, following their respective solubility/saturation and kinetics.
(viii) Authigenic silicon (AuSi) precipitates (e.g., early reverse weathering processes) when DSi porewater concentrations exceed the AuSi saturation threshold.
(ix) ASi and BSi dissolution, and AuSi precipitation rates constants decrease exponentially with depth. The kinetics of each of these diagenetic processes remain (as much as possible) unchanged along the transect.

The sedimentation rates attenuate from fjord head to fjord opening. Sediment biomixing (bioturbation and nonlocal bioirrigation) occur in the top 10 cm depth. Sediment biomixing stops below 10 cm depth. We ran the model until the benthic silicon dynamics reached steady state. The model runs followed the assumptions highlighted above (see i–ix) and appropriate boundary condition (see Table T1) and model parameters (see Table T2 and T3). Note that all three cores are assumed to have core top waters that are saline (Table T2) given that fresher waters are likely to be more buoyant and found further up in the water column. Further details on the model reaction network are provided elsewhere (Ward et al., 2022; Ng et al., 2022).

From our model outputs, we calculated DSi total benthic fluxes along the hypothetical fjord transect and quantified the contribution of each transport process (diffusion, bioturbation, bioirrigation and advection) to these benthic fluxes. Details on flux calculations are provided elsewhere (Ward et al., 2022; Ng et al., 2022).

Differences with published model setups

The main difference between this model and those in (Ward et al., 2022; Ng et al., 2022) is in the parametrization of FeSi reactions. In both previous papers, these reactions were data-driven and implemented implicitly as a desorption only (Ward et al., 2022) and adsorption/desorption (Ng et al., 2022) factor. Here, the reactions are more explicit, accounting for i) a rate constant each that can be treated as constant or have an exponential decay; ii) a fractionation factor that can be changed to better explore how FeSi dynamics impacts $\delta^{30}Si_{DSi}$; and iii) links to DSi production/consumption. Our new model also has a better control of the depth zonation of where adsorption and desorption occur.

Results from the thought experiment

Our model exercise allowed us to recreate a transect along a hypothesised fjord system (see Figure S2). The model results allowed us to assess how ASi glacial supply and FeSi adsorption and desorption processes impact benthic silicon dynamics. Importantly, we also quantified the processes governing porewater DSi production and consumption in the uppermost seafloor depths.

We prescribed a set of conditions along the hypothetical fjord system transect that have strong impacts on the benthic silicon diagenetic dynamics (see Tables T1–T3). Firstly, we imposed an overall attenuation of the terrestrial/glacial supply away from the fjord head (where we defined the position of Core A). Such attenuation resulted in a decrease in sedimentation rates and supply of ASi and (pre-formed) FeSi. Secondly, we assumed an increase in BSi input from fjord head to opening. Altogether, these conditions resulted in a gradual shift in porewater DSi concentrations.

The DSi porewater profiles shared similar trends. Along the transect, DSi porewater concentrations rapidly increased in the uppermost sediment layers. Then, DSi porewater concentrations became more stable and invariant at greater depths. However, the magnitude at which DSi porewater concentration profiles developed differed from the fjord head (Core A) to the fjord opening (Core C). DSi porewater concentrations at the subsurface increased from about 100 μM at the fjord head to greater than 300 μM at the fjord opening. Similarly, in deeper sediments DSi concentrations ranged from 200 μM in Core A to more than 400 μM in Core C. The sharp increase in DSi porewater concentrations near the sediment–water interface (SWI) originated from the dissolution of BSi and ASi. ASi dissolution rates decreased along the transect. Such decrease resulted from low ASi solubility (see Table T3) and, most critically, from the diminished ASi supply (see Table T1). Meanwhile, BSi displays substantially higher solubility (see Table T3). Furthermore, BSi input at the SWI increased downstream (see Table T1) along the fjord. Consequently, greater BSi dissolution occurred, albeit BSi dissolution rates progressively attenuated downcore.. Despite the continuous BSi dissolution at depth, DSi porewater concentrations never reached BSi-sat. In our hypothetical fjord system, AuSi precipitation prevented further DSi accumulation in deeper porewater. Notably, AuSi exhibited large precipitation rates when DSi porewater sustained extensive supply.

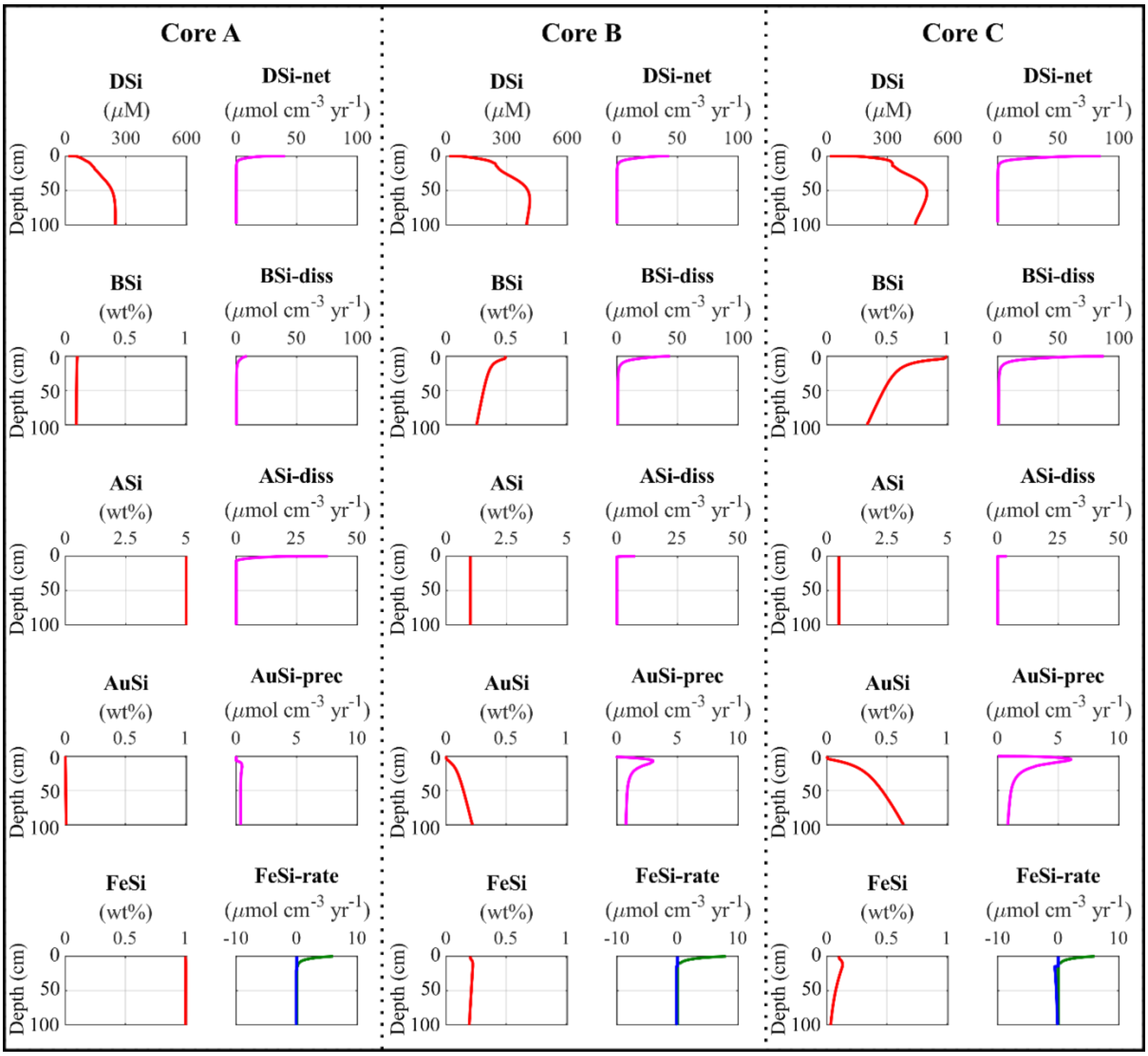

Supplementary Figure S2: Hypothetical fjord system transect model results. Red lines denote silicon species concentrations. Purple lines indicate reaction rates. For FeSi only, green lines indicate FeSi formation rates and blue lines indicate FeSi dissolution rates.

Benthic fluxes

The calculated benthic DSi fluxes progressively increase along the fjord transect (Table T2), reflecting the increasingly larger BSi dissolution rates away from the fjord head.

Supplementary Table T1. Model boundary conditions for the hypothetical fjord system transect

Description	Symbol	Unit	Inner Core A	Middle Core B	Outer Core C	Comments
Dissolved Silicon	[DSi]	μM	15	15	15	[DSi] assumes *typical* fjord bottom water concentration
Biogenic Silicon	[BSi]	wt%	0.1	0.5	1.0	Diatom growth away from high-turbidity zone
Amorphous Silicon	[ASi]	wt%	5.0	1.0	0.5	High input near fjord head and attenuates downstream
Authigenic Silicon	[AuSi]	wt%	$1.0 \cdot 10^{-4}$	$1.0 \cdot 10^{-4}$	$1.0 \cdot 10^{-4}$	Clay formation assumes non-zero value
Fe-bound Silicon	[FeSi]	wt%	1.0	0.2	0.1	Pre-formed FeSi in the water column / glacial transport

Supplementary Table T2. Model transport parameters for the hypothetical fjord system transect

Description	Symbol	Unit	Value	Comments
Run time		yr	30 (A)<br>300 (B)<br>1500 (C)	Reflects the length of model and domain sedimentation rate to achieve for steady state
Model domain	z	cm	100.4	Covers the top 1 metre of sediment - most diagenetically active layer
Temperature	TC	°C	5.0	Assumed bottom water temperature
Salinity	S		31	Assumed bottom water salinity
Depth	SFD	m	100	Assumed seafloor depth
Sedimentation Rate	ω_0	cm yr^{-1}	10 (A)<br>1.0 (B)<br>0.5 (C)	Assumed sedimentation rate attenuation from fjord head to mouth
Bioturbation rate	D_{bio}	cm^2 yr^{-1}	27	Water depth-dependent rate
Bioturbation depth	z_{bio}	cm	10	Global average depth
Porosity - initial	Φ_0		0.7	Assumed
Porosity - final	Φ_z		0.5	Assumed
Porosity - attenuation	Φ_{att}	cm^{-1}	0.1	Assumed
Bioirrigation rate	α_0	yr^{-1}	300	Water depth-dependent rate
Bioirrigation attenuation	α_{att}	cm^{-1}	1.75	Bioirrigation attenuates to about 10 cm depth

* (A), (B), and (C) denote site-specific condition.

Supplementary Table T3. Model reaction parameters for the hypothetical fjord system transect

Description	Symbol	Unit	Values	Comments
BSi-dissolution rate	k-diss		$1.0 \cdot 10^{-1}$	Assuming a "high" reactivity BSi – no changes downstream
BSi-saturation	BSi-Sat	μM	800	BSi-dis = 0 if DSi > 800 μM
BSi dissolution rate attenuation	a-d		0.20	Exponential decrease of k-diss
BSi dissolution rate attenuation minimum	b-d		0.05	Exponential decrease of k-diss
ASi-dissolution rate	k-terdis		$1.0 \cdot 10^{-2}$	Assuming a "high" reactivity ASi – no changes downstream
ASi-saturation	ASi-Sat	μM	100	ASi-dis = 0 if DSi > 100 μM
ASi dissolution rate attenuation	a-t		0.20	Exponential decrease of k-terdis
ASi dissolution rate attenuation minimum	b-t		0.05	Exponential decrease of k-terdis
AuSi-precipitation rate	k-prec		$5.0 \cdot 10^{-6}$	Imposed reverse weathering rate – no changes downstream
AuSi-saturation	AuSi-Sat	μM	100	AuSi-prec = 0 if DSi < 100 μM
AuSi precipitation rate attenuation	a-p		0.10	Exponential decrease of k-prec
AuSi precipitation rate attenuation minimum	b-p		0.05	Exponential decrease of k-prec
FeSi-uptake rate	k-upFeSi		$6.0 \cdot 10^{-6}$ (A)<br>$8.0 \cdot 10^{-6}$ (B)<br>$6.0 \cdot 10^{-6}$ (C)	Imposed FeSi uptake to account for sediment DSi removal by reactive-Fe
FeSi uptake rate attenuation	a-up		0.30	Exponential decrease of k-upFeSi
FeSi uptake rate attenuation minimum	b-up		0.01	Exponential decrease of k-upFeSi
FeSi-release rate	k-reFeSi		$1.0 \cdot 10^{-4}$ (A)<br>$1.0 \cdot 10^{-3}$ (B)<br>$5.0 \cdot 10^{-3}$ (C)	Imposed FeSi release to account for sediment DSi input by reactive-Fe
FeSi release rate attenuation	a-re		0.0	No exponential decrease
FeSi release rate attenuation minimum	b-re		1.0	No exponential decrease
Lower depth FeSi uptake	x-upFeSi	cm	15 (A)<br>10 (B)<br>10 (C)	Lower limit of FeSi uptake – above reactive iron utilisation
Upper depth FeSi release	x-reFeSi	cm	20 (A)<br>15 (B)<br>15 (C)	Upper limit of FeSi release – zone of reactive iron utilisation
Lower depth FeSi release	x-maxFeSi	cm	100	Lower limit of FeSi release

Supplementary References:

1 Pickering, R. A. *et al.* Using stable isotopes to disentangle marine sedimentary signals in reactive silicon pools. *Geophysical Research Letters* **47**, e2020GL087877 (2020).

2 Michalopoulos, P. & Aller, R. C. Early diagenesis of biogenic silica in the Amazon delta: alteration, authigenic clay formation, and storage. *Geochimica et Cosmochimica Acta* **68**, 1061-1085 (2004).

3 Ward, J. P. *et al.* Stable silicon isotopes uncover a mineralogical control on the benthic silicon cycle in the Arctic Barents Sea. *Geochimica et Cosmochimica Acta* **329**, 206-230 (2022).

4 Ng, H. C. *et al.* Benthic dissolved silicon and iron cycling at glaciated Patagonian fjord heads. *Global biogeochemical cycles* **36**, e2022GB007493 (2022).

5 Wang, T. *et al.* Silicon isotopes reveal the impact of fjordic processes on the transport of reactive silicon from glaciers to coastal regions. *Chemical Geology* **670**, 122403 (2024).

6 Huang, T.-H. *et al.* Separating Si phases from diagenetically-modified sediments through sequential leaching. *Chemical Geology* **637**, 121681 (2023).

7 DeMaster, D. J. The supply and accumulation of silica in the marine environment. *Geochimica et Cosmochimica acta* **45**, 1715-1732 (1981).

8 Zhu, X., Hopwood, M. J., Laufer-Meiser, K. & Achterberg, E. P. Incubation experiments characterize turbid glacier plumes as a major source of Mn and Co, and a minor source of Fe and Si, to seawater. *Global Biogeochemical Cycles* **38**, e2024GB008144 (2024).
