# Supplementary material for: Insights into silicon cycling from ice sheet to coastal ocean from isotope geochemistry
Source: Commun Earth Environ. 2025 Apr 19;6(1):305. doi: 10.1038/s43247-025-02264-7 (PMC12009215; doi:10.1038/s43247-025-02264-7)
Supplement: Supplementary file 3 — Description of additional supplementary data [file 43247_2025_2264_MOESM3_ESM.docx]

Description of additional supplementary file

File name: Supplementary Data 1
Description: This data set presents measurements of reactive silicon (Si) from operationally-defined pools isolated using sequential extraction from glacial fjord sediments. Sediments were collected from Kongsfjorden, Svalbard, as part of the Natural Environment Research Council project BIOPOLE (grant no. NE/W004933/1) and from fjords and coastal locations off Iceland as part of the BELSPO funded project DEHEAT. Sediment reactive silica was leached using a sequential extraction method from Michalopoulos and Aller (2004, doi:10.1016/j.gca.2003.07.018) and Pickering et al. (2020, doi:10.1029/2020GL087877): weak-acid leached (Si-HCl), weak-alkaline leached (Si-Na2CO3) and strong-alkaline leached (Si-NaOH). See Supplementary Methods for a full description of approach. Data are used in Figure S1.
